# Involvement of POLA2 in Double Strand Break Repair and Genotoxic Stress

**DOI:** 10.3390/ijms21124245

**Published:** 2020-06-15

**Authors:** Tuyen T. Dang, Julio C. Morales

**Affiliations:** 1 Department of Neurosurgery, University of Oklahoma Health Science Center, Oklahoma City, OK 73104, USA; Tuyen-Dang@ouhsc.edu; 2 Stephenson Cancer Center, University of Oklahoma Health Science Center, Oklahoma City, OK 73104, USA

**Keywords:** POLA2, homologous recombination (HR), non-homologous end-joining (NHEJ)

## Abstract

Cellular survival is dependent on the efficient replication and transmission of genomic information. DNA damage can be introduced into the genome by several different methods, one being the act of DNA replication. Replication is a potent source of DNA damage and genomic instability, especially through the formation of DNA double strand breaks (DSBs). DNA polymerase alpha is responsible for replication initiation. One subunit of the DNA polymerase alpha replication machinery is POLA2. Given the connection between replication and genomic instability, we decided to examine the role of POLA2 in DSB repair, as little is known about this topic. We found that loss of POLA2 leads to an increase in spontaneous DSB formation. Loss of POLA2 also slows DSB repair kinetics after treatment with etoposide and inhibits both of the major double strand break repair pathways: non-homologous end-joining and homologous recombination. In addition, loss of POLA2 leads to increased sensitivity to ionizing radiation and PARP1 inhibition. Lastly, POLA2 expression is elevated in glioblastoma multiforme tumors and correlates with poor overall patient survival. These data demonstrate a role for POLA2 in DSB repair and resistance to genotoxic stress.

## 1. Introduction

Accurate transmission of genetic material from mother cell to each daughter cell is essential for cellular survival. This propagation of genetic material requires the efficient and timely replication of DNA in order to ensure that there are no alterations in the genome of subsequent cell generations. DNA replication is a multistep process that includes initiation and elongation, each with their own machinery [1]. DNA polymerase α (POLα) is required for DNA replication initiation [2,3]. The POLα replication complex, which is made up of two subunits POLA1 (catalytic subunit) and POLA2 (regulatory subunit), has low replication fidelity and lacks proof-reading activity [4]. The POLα complex interacts with primase, to form the primosome [2,3]. The primosome, through the combined functions of primase and POLα, synthesizes a short RNA:DNA primer, that can act as a substrate for the high-fidelity DNA polymerases, POLδ and POLε, to use for processive elongation on the leading and lagging strands of DNA [2,5].

Replication stress is a potent source of genomic instability and leads to an increase in DNA damage, especially double strand breaks (DSBs) [6]. The repair of these replication-induced DSBs is imperative to avoid disease states such as cancer [6]. DSBs are typically repaired by one of two major pathways: homologous recombination (HR) and non-homologous end-joining (NHEJ). Unlike POLδ [7,8] and POLε [9,10,11], little is known about the connection between the DNA damage response and POLα. While there is no reported connection between POLe and DSB repair, loss of POLδ results in defective HR repair and sensitizes cells to PARP1 inhibition [12]. POLδ functions in conjunction with the PIF1 helicase to synthesize DNA at RAD51-mediated D loops [13]. Loss of POLA1 is lethal when combined with a small molecule inhibitor of CHK1 in lung and colorectal cancer cells [14]. Elevated expression of POLA2 has been observed in patients with platinum-resistant mesothelioma [15]. However, there is no direct evidence for a role of POLα in the DNA damage response.

To gain a better understanding of the connection between POLα and DSB repair, we examined cells depleted of POLA2. POLA2-deficient cells display an increase in 53BP1 foci formation, indicative of an increase in endogenous DSBs. Loss of POLA2 also inhibited 53BP1 foci regression after etoposide treatment and both NHEJ and HR repair efficiency. Cells lacking POLA2 also display decreased cellular survival in response to ionizing radiation (IR), etoposide (etp) and the PARP1 small molecule inhibitor, Niraparib. Lastly, we observe that POLA2 is elevated in glioblastoma multiforme (GBM) patient samples and this elevated expression correlated with poor overall survival.

## 2. Results

### 2.1. Elevated POLA2 Expression Is Detrimental to GBM Patients

**Figure 1:**
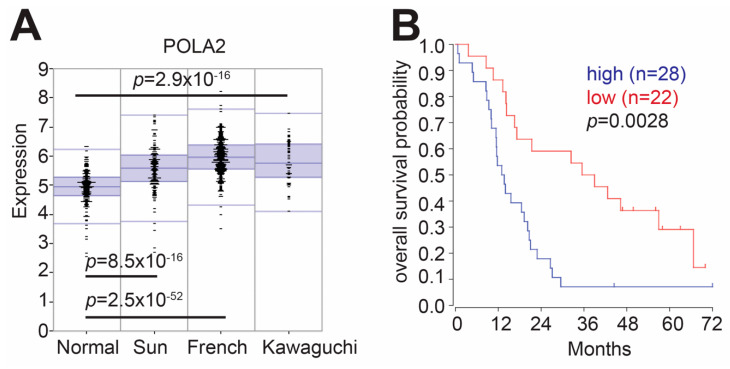
Elevated POLA2 expression correlates with poor overall glioma patient survival. (**A**) Expression of POLA2 in normal and tumor datasets. (**B**) XRN2 high versus XRN2 low overall patient survival is publicly available through the R2 Genomic and visualization platform. Default settings provided by the platform were used.

POLα is comprised of two subunits: POLA1 (catalytic subunit) and POLA2 (regulatory subunit) [4]. Using the R2: Genomics Analysis and Visualization Platform (http://r2.amc.nl), we examined microarray data from GBM patients provided by the Kawaguchi (GEOID: gse43378) [16], Sun (GEOID: gse4290) [17] and French (GEOID: gse16011) [18] datasets, and found that POLA2 expression is elevated in GBM tumor samples, as compared to normal brain tissue (Figure 1A). Using the Kawaguchi dataset, we found that elevated POLA2 expression also correlated with poor overall survival of GBM patients as compared to patients with lower POLA2 expression (Figure 1B). POLA1 expression is also elevated in these datasets (Supplemental Figure S1A), yet there is no significant difference in overall patient survival (Supplemental Figure S1B). As elevated POLA2 expression correlated with poor overall survival, while POLA1 did not, we focused the remainder of this study on POLA2.

### 2.2. Loss of POLA2 Leads to Increased Spontaneous DSB Formation and Impairs DSB Repair Rates

**Figure 2:**
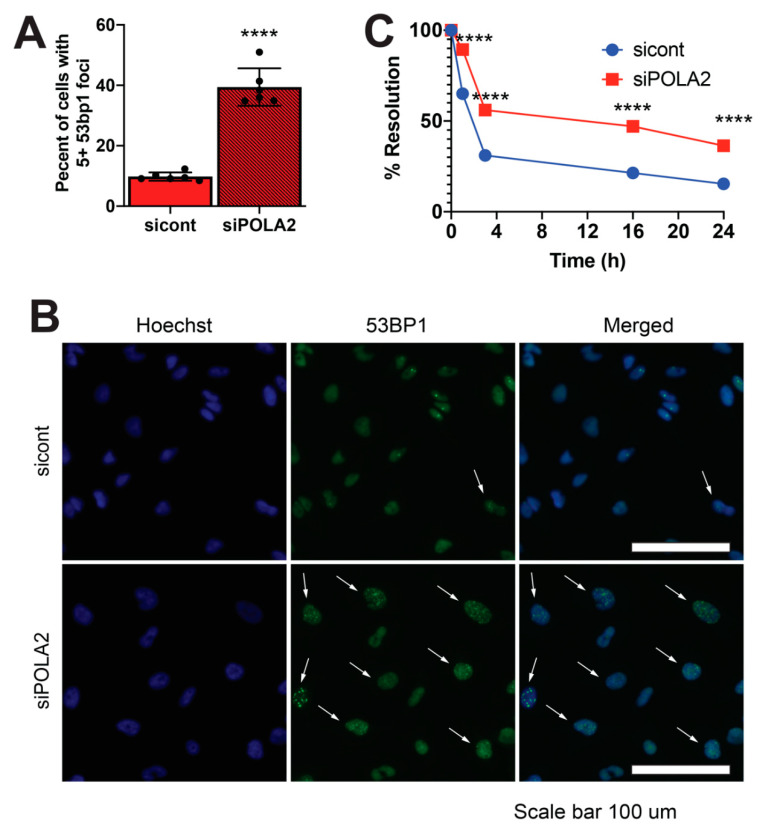
POLA2-deficient cells demonstrate increased spontaneous 53BP1 foci formation and delayed DNA repair kinetics. (**A**,**B**) 53BP1 foci formation was measured in LN229 cells exposed to POLA2 and control (cont) siRNAs by immunofluorescence using the Cytation5 from Biotek. Gen5 software was used to automate 53BP1 foci quantitation. (**C**) 53BP1 foci disappearance was monitored in LN229 cells exposed to cont and POLA2 siRNAs. Statistical analysis was performed using student’s *t* test. **** *p* < 0.00001. Experiments were performed in triplicate with at least 1000 cells counted for each condition in each experiment. White arrows depict cells with +5 53BP1 foci.

To gain a better understanding of the potential role of POLA2 in DSB repair, we ablated POLA2 expression in LN229 and U251 cells, two established human GBM cell lines, using three unique POLA2 siRNAs (Supplemental Figure S2A,B). Using immunofluorescence, we imaged 1000 LN229 cells. We found that loss of POLA2 led to an increase in cells displaying 5+ 53BP1 foci. Roughly 45% of cells in an asynchronous population treated with POLA2 siRNAs displayed 5+ 53BP1 foci, as compared to ~10% of cells treated with control siRNA (Figure 2A,B). These data suggest that loss of POLA2 results in an increased amount of spontaneous DSB formation.

While 53BP1 foci formation is a marker of DSB formation, resolution of 53BP1 foci can be used as an indication of DSB repair [19,20]. We exposed LN229 cells with and without POLA2 with 5 μM of etoposide (etp) for 30 min to induce DSB formation. We then examined these cells for 53BP1 foci at 0, 1 and 3 h post etp exposure. The percentage of cells displaying 5+ 53BP1 foci in LN229 lacking POLA2 was 89% and 56% at 1 and 3 h after etp exposure as compared to 65% and 31% in control cells (Figure 2C). Consistent with these data, we found that loss of POLA2 also slows down the disappearance of 53BP1 in U251 cells (Supplemental Figure S3B). The delay in 53BP1 foci regression is often times associated with defects in NHEJ and HR DSB repair pathways [19,20,21].

### 2.3. POLA2 Loss Sensitizes Cells to Genomic Insult

**Figure 3:**
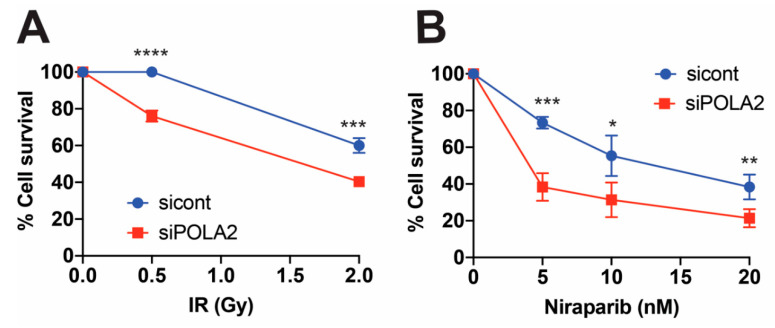
Cells lacking POLA2 display increased sensitivity to ionizing radiation (IR) exposure and PARP1 inhibition. Cellular survival was measured in LN229 cells exposed to control (cont) and POLA2 siRNAs. These cells were then treated with (**A**) ionizing radiation (IR) or (**B**) Niraparib, a PARP1 small-molecule inhibitor, at indicated doses. Statistical analysis was performed using student’s *t* test. * *p* < 0.05, ** *p* < 0.01, *** *p* < 0.001 and **** *p* < 0.0001.

Along with slowed DSB repair kinetics, loss of the NHEJ and HR repair pathways typically sensitizes cells to exogenous genomic insults, such as ionizing radiation (IR) [22,23,24,25]. In addition, loss of the HR repair pathway also sensitizes cells to PARP1 inhibition [26]. To examine whether loss of POLA2 sensitizes cells to exogenous genomic stress, we exposed POLA2 lacking LN229 and control cells to IR and Niraparib, a PARP1 small-molecule inhibitor [27]. Loss of POLA2 led to a decrease in the surviving fraction in LN229 POLA2-deficient cells exposed to ionizing radiation (IR) and Niraparib (Figure 3A,B). Loss of POLA2 also led to increased sensitivity to Niraparib and etp in U251 cells (Supplemental Figure S4A,B).

### 2.4. Loss of POLA2 Inhibits HR and NHEJ Repair

**Figure 4:**
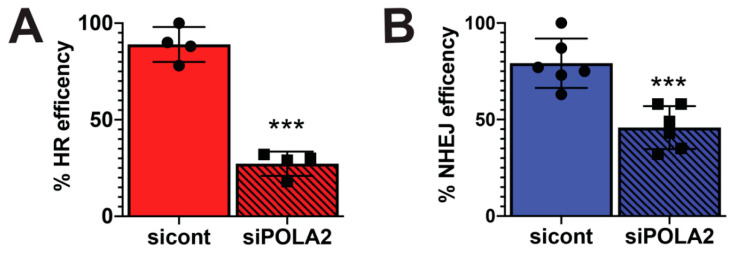
Loss of POLA2 impairs NHEJ and HR repair efficiency. (**A**) Homologous recombination (HR) or (**B**) non-homologous end-joining (NHEJ) utilization was measured in the DR and EJ5 U2OS reporter cell lines exposed to control (cont) or POLA2 siRNAs. For each experiment, NHEJ and HR repair efficiency was determined by counting 10,000 cells per condition and normalizing to mock treated cells. Statistical analysis was performed using a student’s *t* test. *** *p* < 0.001.

To determine if loss of POLA2 adversely affects DSB repair, we used the EJ5 and DR U2OS GFP reporter cells. The DR reporter cell line was used to measure how loss of POLA2 affects HR repair, while the EJ5 cell line was used to measure total NHEJ repair [28]. Both these cell lines contain an expression cassette for green fluorescent protein (GFP), that is interrupted by an IsceI endonuclease restriction site. Expression of the IsceI endonuclease generates a DSB that is repaired by NHEJ or HR depending on what cell line is used. GFP expression is used to determine a successful repair event. DR and EJ5 cells were transfected with control and POLA2 siRNAs. We found that the relative percentage of GFP expressing cells in DR and EJ5 cells without POLA2 was 27% and 45%, respectively (Figure 4A,B). These data demonstrate a 70% and 35% loss in HR and NHEJ repair efficiency when POLA2 is lost. These data are consistent with the observation that 53BP1 foci is retained for longer periods of time in POLA2 lacking cells as compared to control cells and clearly demonstrates a role for POLA2 in DNA repair.

## 3. Discussion

The response and repair of genomic lesions, in particular DSBs, is critical to cellular survival and avoiding possible disease states such as cancer. Thus, uncovering cellular factors important in DSB repair is imperative to understanding how genomic stability is maintained. Our data is the first to provide evidence for POLA2 in promoting DSB repair and protection against genotoxic stress.

### 3.1. Connecting POLA2 and the DNA Damage Response

POLA2 is a POLα subunit, which is involved in the initiation of DNA replication [1,2,3]. After unwinding of the parental DNA, primase synthesizes a short RNA primer that is extended by POLα to create a DNA primer that will be utilized by POLδ and POLε for progressive DNA elongation [2,3]. POLε and POLδ requires this DNA component of the short RNA:DNA primer for progressive DNA extension [2,5]. It is possible that the absence of POLA2 impairs POLε and POLδ‘s ability to extend DNA, leaving a DNA bubble with an RNA component. These types of DNA bubbles can then be processed into DSBs through the action of XPF and XPG and engagement of the nucleotide excision repair pathway [29]. Thus, the increase in spontaneous 53BP1 foci observed in POLA2-deficient cells (Figure 2A) can be explained by a lack of DNA extension after DNA unwinding at the origin of replication. Yet, this mechanism for 53BP1 foci formation does not explain why the disappearance of 53BP1 foci is delayed after exogenous DNA damage (Figure 2B) and the increased sensitivity to genotoxic stress (Figure 4).

One key piece of data connecting POLA2 to DNA repair is the decreases in cellular survival after genotoxic stresses (Figure 3). Cells lacking POLA2 demonstrated increased sensitivity to Niraparib, a PARP1 small-molecule inhibitor [27]. The sensitivity to PARP1 inhibition is commonly associated with a defective HR repair pathway [26]. In addition, we found that loss of POLA2 also sensitizes cells to IR, a hallmark characteristic of DSB repair failure, whether it is loss of HR or NHEJ repair [19,20,21,23,24]. These data suggest a more functional role in DSB repair for POLA2.

The delay in DSB foci repair and increased sensitivity to DNA damaging agents is often connected to a functional loss in the NHEJ or HR repair pathways [19,20,21]. Interestingly, we found that loss of POLA2 impaired both NHEJ and HR repair pathways. This is an important piece of data due to the fact that the NHEJ repair pathway is functional throughout the cell cycle, while HR repair is restricted to S and G2 [30]. The impairment in NHEJ repair suggests that the requirement for POLA2 in DSB repair is cell cycle-independent.

### 3.2. POLA2 as a Therapeutic Target

Glioblastomas (GBMs) are one of the most deadly types of neoplasms known and are associated with one of the worst five year survival rates for patients [31]. The first-line regimen for treating GBM patients is surgical resectioning followed by radiation, and even with improvement in the GBM therapeutic regimen, the five year survival of GBM patients has not changed in over 50 years [32]. One reason for such low survival rates in GBM patients is the rate of recurrent malignancies that is associated with therapeutic resistance [33,34]. A key factor in GBM therapeutic resistance, whether it is acquired or intrinsic, is upregulation of the DNA repair factors [35,36]. We found that POLA2 expression is elevated in glioma patients and this elevation is associated with poor overall survival (Figure 1). Our data demonstrate that loss of POLA2 results in increased sensitivity to IR and Niraparib (Figure 4), IR being one of the standard GBM therapeutic modalities and Niraparib being the subject of two active and one completed clinical trial (clinicaltrials.gov). This suggest that targeting POLA2 specifically or the POLα activity may enhance current GBM therapeutic regimens.

## 4. Materials and Methods

Gene expression and overall survival probability: Gene expression analysis was performed using the R2 genomics analysis and visualization platform (http://r2.amc.nl). Default settings were used to compare expression of POLA1 and POLA2 in a set for normal brain samples (n = 172, Berchtold set) to adult glioma samples (n = 153, Sun set; n = 40, Kawaguchi set; n = 284, French set). Overall survival probability for POLA1 and POLA2 was determined using default settings applied to the Kawaguchi dataset.

Cell culture: LN229, U251 and U2OS were maintained in 5% CO2 at 37 °C. LN229 were cultured in 5% fetal bovine serum (FBS) in high-glucose L-glutamine containing DMEM (DMEM) supplemented with 1X penicillin/streptomycin (Pen/Strep). U251 were cultured in 10% FBS in Minimum Essential Medium Eagle supplemented with a final concentration of 1 mM L-glutamine, 1 mM Sodium pyruvate, and 1X Pen/Strep. U2OS were cultured in 10% FBS in DMEM with 1X Pen/Strep.

Western blot: Immuno-blotting was carried out as previously described [37]. Briefly, cells were lysed in RIPA buffer and supplemented with protease inhibitors. After addition of Laemmli sample buffer, samples were run on a 10% SDS-Page gel. Samples were transferred onto a PVDF membrane via BioRad’s (Hercules, CA, USA) semi-dry apparatus. Membranes were blocked in 0.01% Casien block for 1 h and incubated with antibodies at 4 °C overnight (O/N). Membranes were then washed with TBST buffer, probed with secondary antibodies, washed with TBST buffer and then imaged on a BioRad Chemidoc MP. Primary antibodies used were POLA2 (Invitrogen, cat no. PA5-58015, 1:500) and b-actin (ThermoFisher Waltham, MA, USA), cat no. MA5-15739). Secondary antibodies used were Alexa 488 (ThermoFisher, cat no. A11008) and Alexa 680 (ThermoFisher cat no. A21057).

Immunofluorescence: Immunofluorescence was carried out as previously described [37]. Cells were fixed with 2% formaldehyde in PBS and permeabilized with 0.5% TritonX-100. Cells were blocked with 10% serum in immunofluorescence buffer (IF buffer) (PBS, 1% BSA, 0.2% TritonX-100 and 0.05% Tween-20) for 1 h and then incubated with antibodies O/N. Samples were washed 3X in IF buffer, probed with 2° antibodies, washed 3X in IF buffer and stored in PBS at 4 °C until imaging. Antibody used was 53bp1 (ThermoFisher, cat no. PA1-16566, 1:1000) and was counterstained with Hoechst (ThermoFisher, cat no. H3570, 1:2000)

I-SecI-based DNA repair assays: Assays were carried out as described with slight modifications [28]. Cells were reverse transfected with 50 nM of siRNA and Mission siRNA transfection reagent (Sigma Aldrich, cat no. S1452-1ML) in a 96-well plate for 24 h, then I-SecI plasmid (60 ng/well) was transfected into the cells, with no additional amount of siRNA needed. Media was replaced 24 h after plasmid transfection. 72 h post-plasmid transfection, cells were fixed with 2% formaldehyde in PBS and counterstained with Hoechst. Cells were imaged for Hoechst and GFP signals. Percent efficiency was determined by percentage of cells positive for GFP and Hoechst (EJ5-GFP and DR-GFP).

Cellular survival studies: 400–500 cells were reverse transfected with 0.5 nM of siRNA and siRNA transfection reagent per well in a 96-well plate for 24 h. Cells were drug-treated for ~17 h, washed with 1X PBS, and then fed with fresh growth media. For irradiation studies, cells were irradiated ~24 h post-transfection on an X-RAD 320 irradiator. Drug-treated or irradiated plates were then harvested for Hoechst staining and intensity reading on the Cytation 5, 7–8 days post-transfection. Relative survival was determined by non-drug or non-irradiated-treated readings.

Imaging/Fluorescent intensity: Imaging was done on a BioTek’s (Winooski, VT, USA) Cytation 5 imager. The following objectives were used: 10X phase Plan Fluorite WD 10 NA 0.3 and 20X phase Plan Fluorite WD 6.7 NA 0.45. Spot counting is a propriety application within Gen5 (BioTek). Fluorescent intensity readings were conducted on the BioTeK Cytation 5 plate reader, monochrome setting.

siRNAs: sicont, Dharmacon (Lafayette, CO, USA), cat no. D-001810-10-05, UGGUUUACAUGUCGACUAA, UGGUUUACAUGUUGUGUGA, UGGUUUACAUGUUUUCUGA, UGGUUUACAUGUUUUCCUA.

siPOLA2, Sigma Aldrich (St. Louis, MO, USA),
SASI_Hs02_00334255, CUAUGAGUCGUUCUAUGUU
siPOLA2, Sigma Aldrich (St. Louis, MO, USA),
SASI_Hs01_00177968, CAUUCAAGAGCUAAUUGAA
siPOLA2, Sigma Aldrich (St. Louis, MO, USA),
SASI_Hs01_00177970, CUGAACAACAAGUCAGUGA

## Supplementary Materials

Supplementary figures and materials can be found at https://www.mdpi.com/1422-0067/21/12/4245/s1.
