# Supplementary material for: Involvement of POLA2 in Double Strand Break Repair and Genotoxic Stress
Source: Int J Mol Sci. 2020 Jun 15;21(12):4245. doi: 10.3390/ijms21124245 (PMC7352189; doi:10.3390/ijms21124245)
Supplement: Supplementary file 1 [file ijms-21-04245-s001.pdf]

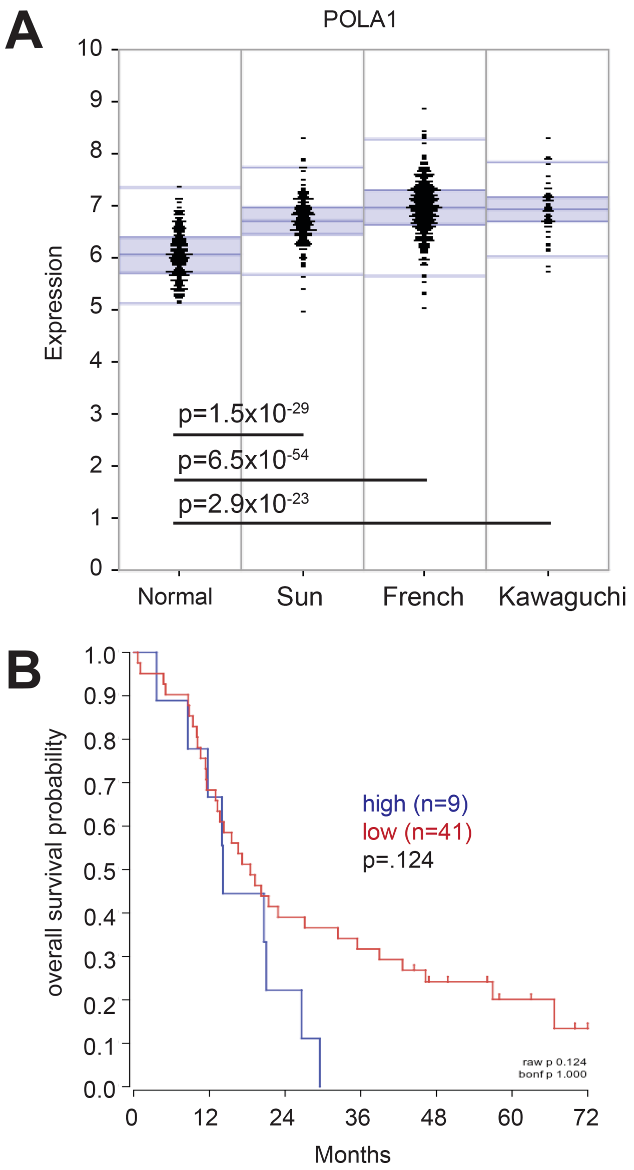

Supplemental Figure S1: POLA1 expression is elevated in gliomas but does not correlate with poor overall survival patient survival. **A and B**. Expression of POLA1 in normal and tumor datasets and overall patient survival is publicly available through the R2 Genomic and visualization platform. Default settings provided by platform was used.

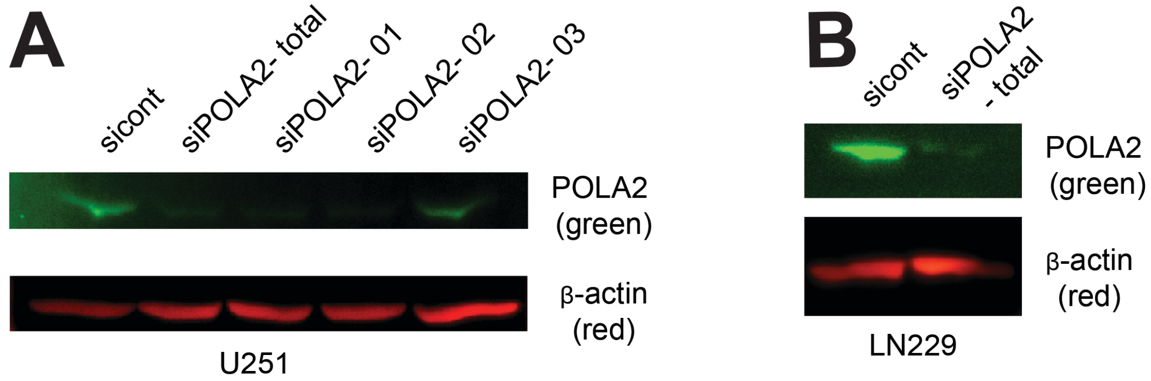

Supplemental Figure S2: Confirmation of POLA2 loss in LN229 and U251 cells. Steady-state protein levels of POLA2 in U251 and LN229 cells exposed to control or POLA2 siRNAs (pooled or individual) was measured by western blot.

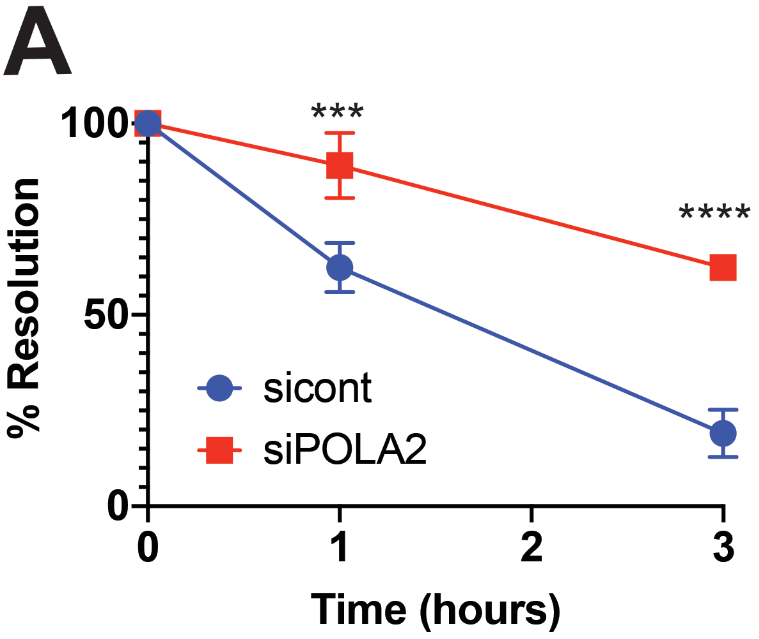

Supplemental Figure S 3: Loss of POLA2 results in delayed 53BP1 foci regression. 53BP1 foci disappearance was monitored in U251 cells exposed to control (cont) and POLA2 siRNAs by immunofluorescence using the Cytation5. Gen5 software automated 53BP1 counting. Statistical analysis was performed using students t test. ***=p,.001 and ****=p<.0001. Experiments were performed in triplicate and at least 200 cells were counted for each experiment.

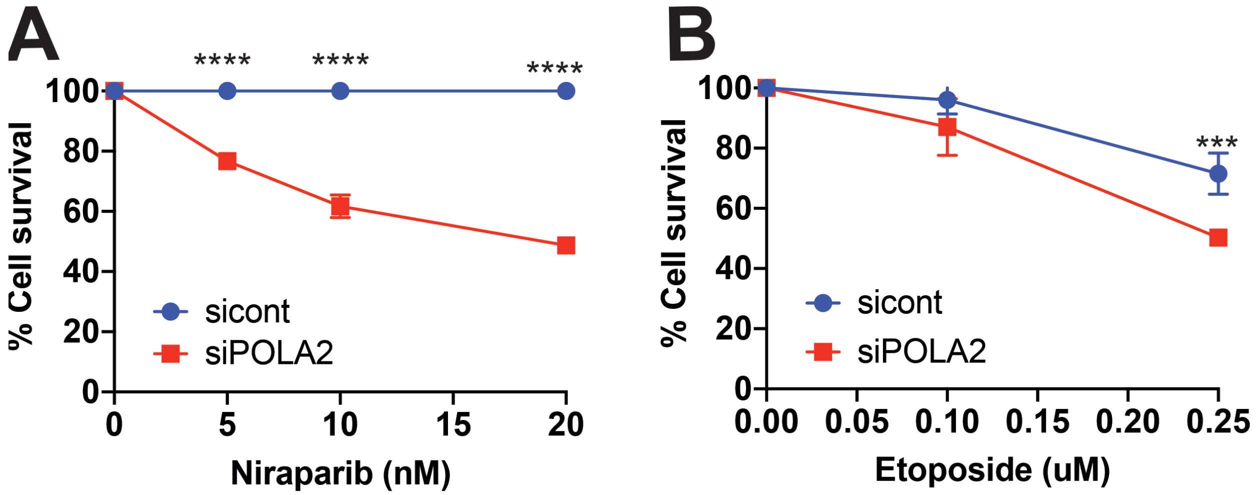

Supplemental Figure S4: Loss of POLA2 sensitizes cells to Etoposide and Niraparib. Cellular survival was measured in U251 cells exposed to control (cont) and POLA2 siRNAs. These cells were either treated with **A.** Niraparib or **B**. Etoposide at indicated doses. Statistical analysis was performed using students t test. ***=p<.001 and ****=p<.0001.
